# Diverse definitions of prolonged labour and its consequences with sometimes subsequent inappropriate treatment

**DOI:** 10.1186/1471-2393-14-233

**Published:** 2014-07-16

**Authors:** Astrid Nystedt, Ingegerd Hildingsson

**Affiliations:** 1 Department of Nursing, Mid Sweden University, Holmgatan 10, 851 70 Sundsvall, Sweden; 2 Department of Women’s and Children’s Health, Obstetrics and Gynaecology, Uppsala University, 751 85 Uppsala, Sweden; 3 Department of Women’s and Children’s Health, Division of Reproductive and Perinatal Health Care, Karolinska Institutet, 171 76 Stockholm, Sweden

**Keywords:** Birth experience, Dystocia, Prolonged labour

## Abstract

**Background:**

Prolonged labour very often causes suffering from difficulties that may have lifelong implications. This study aimed to explore the prevalence and treatment of prolonged labour and to compare birth outcome and women’s experiences of prolonged and normal labour.

**Method:**

Women with spontaneous onset of labour, living in a Swedish county, were recruited two months after birth, to a cross-sectional study. Women (n = 829) completed a questionnaire that investigated socio-demographic and obstetric background, birth outcome and women’s feelings and experiences of birth. The prevalence of prolonged labour, as defined by a documented ICD-code and inspection of partogram was calculated. Four groups were identified; women with prolonged labour as identified by documented ICD-codes or by partogram inspection but no ICD-code; women with normal labour augmented with oxytocin or not.

**Results:**

Every fifth woman experienced a prolonged labour. The prevalence with the documented ICD-code was (13%) and without ICD-code but positive partogram was (8%). Seven percent of women with prolonged labour were not treated with oxytocin. Approximately one in three women (28%) received oxytocin augmentation despite having no evidence of prolonged labour. The length of labour differed between the four groups of women, from 7 to 23 hours.

Women with a prolonged labour had a negative birth experience more often (13%) than did women who had a normal labour (3%) (*P <0.00*). The factors that contributed most strongly to a negative birth experience in women with prolonged labour were emergency Caesarean section (OR 9.0, 95% CI 1.2-3.0) and to strongly agree with the following statement ‘My birth experience made me decide not to have any more children’ (OR 41.3, 95% CI 4.9-349.6). The factors that contributed most strongly to a negative birth experience in women with normal labour were less agreement with the statement ‘It was exiting to give birth’ (OR 0.13, 95% CI 0.34-0.5).

**Conclusions:**

There is need for increased clinical skill in identification and classification of prolonged labour, in order to improve care for all women and their experiences of birthing processes regardless whether they experience a prolonged labour or not.

## Background

Prolonged labour or dystocia is a common birth complication and constitutes the major indication of instrumental deliveries and delivery by emergency Caesarean section (CS) [1,2]. Diagnosing prolonged labour is inherently difficult and it is a controversial issue that has been discussed ever since Friedman introduced the graphic analysis of labour, a study based on 100 women [3]. Using the same criteria and definition of prolonged labour and labour progress for every women, both for nulliparaous and multiparaous has recently been questioned by Laughon and coworkers [4]. In their work of defining different patterns in labour progress they propose an alternative approach to diagnose a slow progress of labour. This challenges the established knowledge and highlights that an accurate diagnosis of prolonged labour is important for evidence based clinical decision making and for women who experience a prolonged labour.

Approximately 8% of all women giving birth are affected by a prolonged labour [5], and the complication occurs three times more often among primiparae than among multiparae in Western countries [6,7]. National statistics show that during the years 1988 to 1998 six percent of Swedish primiparae experienced a prolonged labour. Statistics furthermore show that the prevalence of prolonged labour among all Swedish women giving birth during the years 2001 – 2003 was 14%, with regional variations between 5-36% [8]. The different proportions in prevalence among first-time mothers and all Swedish women giving birth may not reflect the actual prevalence of prolonged labour. It may indicate that the codes of diagnosis and the prevalence of prolonged labour can vary in the different regions in Sweden. In a Swedish study by Selin (2009), a prevalence of prolonged labour was 33% in first-time mothers and 7% in women with previous children [9] and a Danish prospective study of nulliparas, 37% were diagnosed with prolonged labour [10].

Maternal risk factors that increase the risk for prolonged labour include primiparity [11] and total maternal weight gain or high body mass index [12,13]. Foetal risk factors include a heavy birth weight, large head circumference and occiput posterior presentation [14,15]. A prolonged labour is also associated with worse labour pain than expected, leading to greater use of epidural analgesia and risk of operative interventions [10,16].

The progress of labour is documented using partogram aiming for early detection of slow progress and preventing change from a normal labour to a prolonged labour [17]. The most common definition of or diagnostic criteria for prolonged labour is protraction disorders (slower than normal) or arrest disorders (complete cessation of progress) [18]. It is a historical criteria based on previous evidence about progress of labour in the practise of labour management that may no longer be clinically useful [19]. But still the use of oxytocin to treat prolonged labour depends on which diagnostic criteria are used to define a slow progress of labour or a prolonged labour [20].

Early use of oxytocin augmentation and early amniotomy are common interventions used to speed up slow progress and encourage dilation. The existing strategies of oxytocin augmentation can be used to prevent slow progress from occurring, or to accelerate labour if the dilation rates become slower than the accepted minimum rate as defined by the diagnostic criteria. Risk factors that can be associated with the use of oxytocin augmentation are emergency Caesarean section [21], hyper stimulation [22] and for the newborn, a low Apgar score [23].

Bugg (2013) showed that the advantages of the use of oxytocin could be a reduction of length of labour, but its use does not increase the rate of normal births among women with slow progress [24]. Still, a slow progress of labour is one of the leading causes of increasing Caesarean sections. The CS rate in the Western world and in Sweden specifically is steadily increasing despite the increased use of oxytocin for augmentation of labour. Both interventions, an unplanned Caesarean section and the use of oxytocin for augmentation, affect women’s and infants’ health [25,26].

Research on the experiences of women who had high levels of obstetric interventions in general indicates a tie to negative birth experiences. Feelings of stress and psychological trauma following an emergency Caesarean delivery accompany descriptions of anxiety about future motherhood [27,28]. If the experience of giving birth is one of unexpectedly slow or poor progress of labour and, eventually, obstetric interventions, then it will most likely be negatively described [16]. In a case-referent study of prolonged labour Nystedt and co-workers (2005) found that one-third of women with slow progress had a negative birth experience, and two-thirds stated that the experience had marked them for life [29]. A common finding reported is that women with more obstetric interventions and a negative birth experience express greater dissatisfaction both in the short and in the long term [25,30]. For women in the aftermath of a prolonged labour it is of great importance that treatment and care are based on correct classifications and diagnoses of prolonged labour. In clinical practice when diverse opinions exist among midwives and obstetricians about the definition of prolonged labour, and guidelines differ between hospitals, then the clinical decisions about interventions may differ. The disadvantage of variations in identification, treatment and care could influence women’s birth experiences and birth outcomes negatively. In this study, we aimed to explore the prevalence and treatment of prolonged labour and to compare birth outcome and women’s experiences of prolonged and normal labour.

## Methods

### Setting

This study is part of a prospective longitudinal survey conducted at three hospitals with obstetric wards in the County of Vasternorrland in northern part of Sweden during one year 2007-2008. The annual birth rates at the hospitals were approximately 1500, 600 and 300 per year. Women who were recruited were asked to complete a total of four questionnaires: the first during mid-pregnancy (week 17–19), the next in late pregnancy (week 32–34), the third, 2 months after birth, and the last questionnaire was given 1 year after birth. For this study we used the questionnaire which was given 2 months after birth together with socio-demographic background data collected in mid-pregnancy.

The sample of women is representative of the population in terms of background characteristics as the numbers of women included in the sample covers the population in the region of all pregnant Swedish speaking women.

Following local guidelines intrapartum standard care for women in active labour includes midwifery support, encouraging women to walk around, and to eat and drink free. When labour progress slows down amniotomy is the first choice and if it is necessary oxytocin is used for augmentation to speed up the progress Midwives work independently with normal births and when complication occurs then they work in collaboration with an obstetrician. Midwives usually perform all the births, except for the operative ones, though even in these cases, she is still present to take care of the baby and the mother afterwards [31].

### Recruitment

Women who were listed for a routine ultrasound were, two weeks prior to the ultrasound examination, sent a letter of invitation with information about the study. Ultrasound examinations were routinely performed during gestational weeks 17 to 19 and attended by the majority of women. Swedish-speaking women with a normal ultrasound examination were approached by the midwife in charge of the examination, who asked the women if they were willing to participate in the study. A consent form was signed and women who agreed to participate were given the first questionnaire at the ultrasound ward, where they were asked to complete the form and leave it in a sealed envelope. They also had the opportunity to take the questionnaires home and return them in a stamped envelope. Two reminder letters were sent to non-responders after two and four weeks respectively.

The number of women who in mid-pregnancy consented to participate was 1506 and 1212 (80%) returned the first questionnaire, the second was completed by 1042 (70%). Two months after birth, 1242 women were sent the third questionnaire and 936 returned the questionnaire. The sample corresponds to 62% of those who originally consented to participate in the longitudinal survey and 75% of those who received the third questionnaire. For the purpose of this study, women with induction of labour and planned Caesarean section were excluded.

### Data collection

Data was collected by two questionnaires and from electronic birth records. From the first questionnaire, background data (age, civil status, level of education, country of birth, use of tobacco, Body Mass Index and parity) was collected. Two months after birth, women were asked to provide details about self-rated length of labour and birth (in hours), their perceived length of labour (0-7, prolonged to rapid), pain intensity and pain experience (0-7, very positive to very negative). Mode of birth, labour augmentation, use of epidural, self-reported complications during birth, together with a question about the overall experience of birth. The question about the birth experience was responded to on a 5-point Likert scale ranging from ‘Very positive’ to ‘Very negative’. In the analysis, the variable was dichotomised into ‘Positive’ (Very positive, Positive) and ‘Less than positive’ (Mixed feelings, Negative and Very negative). The reason for this dichotomization was based on the skewed nature of the variable. The majority of these questions were used previously in a national Swedish survey (KUB) [32].

Prolonged labour was identified in two ways. First, all birth records were scrutinized manually for medical diagnosis according to the international classification for disease (ICD10) [18], which defines prolonged active phase of labour as:

a) progress of slower than one cm per hour with the following codes (O62, O62.0, O62.1),

b) as irregular or poor uterine contractions with the following codes (O62.4, O62.8, O62.9), as a labour with regular uterine contractions for more than 12 hours (O63, O63.0 O63.9),

c) and/or as a cervical dilation of ten cm for more than three hours (O63.1).

In addition to recording ICD-Codes all partograms within the birth records were checked by the first author, for the progress of labour. If the action line exceeded two hours according to the local hospital guidelines the birth was coded as prolonged. In reporting the use of oxytocin, only records which indicated use for augmentation of labour were included. In the context of this paper, normal labour refers to women who did not have a prolonged labour. Four groups were identified; women with prolonged labour as identified by documented ICD-codes; women with prolonged labour as identified by partogram inspection but no ICD-code; women with normal labour; and women with normal labour augmented with oxytocin.

Ethics approval (Dnr 05-134Ö) was obtained from the regional ethic committee of the Medical Faculty, Umea University, Umea, in northern Sweden and from the Mid Sweden University.

### Analysis

Statistical analyses were conducted using the Statistical Package for Social Sciences, SPSS, version 20.0 (SPSS, Inc., Chicago, USA). Descriptive and inferential statistics were used in the analysis. A one-way ANOVA was used to compare means and standard deviations between the previously defined groups of prolonged and normal labour. Crude and adjusted odds ratios with a 95% confidence interval [33] were calculated between the two groups of prolonged labour with and without diagnosis and between the groups of normal labour, with and without oxytocin augmentation. Finally, logistic regression models were performed in order to reveal factors most strongly associated with a negative birth experience in women with a prolonged labour and in women with normal labour.

## Results

**Figure 1:**
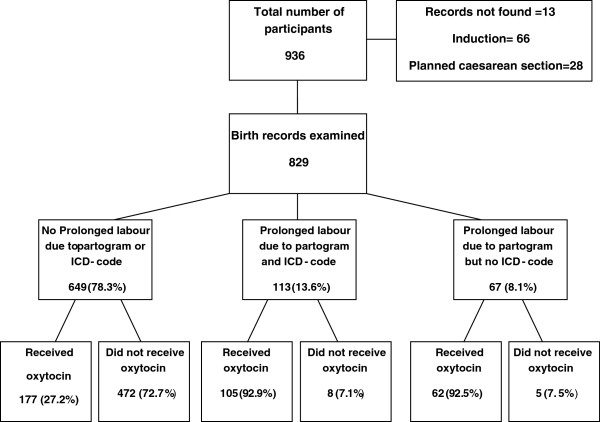
Flowchart of selection procedure.

In total, 936 women responded to the questionnaire two months after birth. Birth records were missing in 13 cases, 28 women were delivered by planned Caesarean section and 66 women were induced, leaving 829 women in the study. Of the 829 remaining women included in the study, 649 were not diagnosed with prolonged labour, 113 had an ICD diagnostic code of prolonged labour also confirmed in the partogram, and 67 did not have an ICD code, but it was evident in the inspection of the partogram that labour was prolonged (Figure 1). In the group of women with a diagnosis of prolonged labour, 7% did not receive treatment. A similar percentage 7.5% was found in group of women without an ICD-code of prolonged labour but evidence as seen on the partogram (Figure 1). In the group of women with normal labour, 27% received augmentation with synthetic oxytocin despite the lack of documented ICD-code or evidence on the partogram of prolonged labour. In both groups of prolonged labor and among those who did not receive oxytocin, there was no information recorded on the reason why they did not receive treatment. The prevalence of prolonged labour in this study was 21.7% for the whole sample. In primiparous women the prevalence was 35.6% and in multiparous women 10.2%.

Table 1 shows the background characteristics of the participating women. The majority were aged 25-35 years, living with their partner and born in Sweden. Rather few women used tobacco and the majority had some university education. The only difference between women with and without prolonged labour was parity, with more primiparas belonging in the prolonged labour group.

**Table 1:** Background characteristics

	**Prolonged labour**^**a, b**^	**Normal**^**a **^**labour**	**Crude odds ratio**
	**n = 180**	**n = 649**	
	**n (%)**	**n (%)**	**(95% CI)**
**Socio-demographic background**			
**Age**			
<25 years	19 (10.6)	84 (13.0)	1.3 (0.8-2.3)
25-35 years	144 (80.4)	469 (72.6)	1.0 Ref.^c^
>35 years	16 (8.9)	93 (14.4)	0.7 (0.4-1.5)
**Previous children**			
Primipara	133 (74.3)	241 (37.3)	4.8 (3.3-7.0)***
Multipara	46 (25.7)	405 (62.7)	1.0 Ref.
**Marital status**			
Living with partner	175 (97.8)	627 (97.1)	1.0 Ref.
Not living with partner	4 (2.2)	19 (2.9)	0.7 (0.2-2.2)
**Country of birth**			
Sweden	175 (97.8)	616 (95.4)	1.0 Ref.
Other country	4 (2.2)	30 (4.6)	0.5 (0.2-1.3)
**Education**			
Elementary school	6 (3.4)	25 (3.9)	1.2 (0.5-3.0)
High school	74 (41.3)	6257(40.3)	1.0 Ref.
College/university	99 (55.3)	355 (55.7)	1.2 (0.5-2.9)
**Tobacco use in mid pregnancy (smoking/snuff)**			
Yes	5 (2.8)	42 (6.5)	0.4 (0.1-1.1)
No	174 (97.2)	604 (93.5)	1.0 Ref.
**Body Mass Index**			
<20	14 (8.3)	71 (11.5)	0.6 (0.3-1.2)
20-25	80 (47.6)	268 (43.2)	1.0 Ref.
25-30	64 (38.1)	248 (40.0)	0.8 (0.5-1.2)
30-	10 (6.0)	33 (5.3)	1.0 (0.4-2.1)

^a^Prolonged labour includes women who experienced a prolonged labour with and without diagnosis.

^b^Number may not add to 100% due to missing internal values.

^c^Reference = Women not exposed to the studied variable.

***p < 0.001.

Length of labour and experience of pain are shown in Table 2. Both groups of women following a prolonged labour reported longer lengths (measured in hours), and they also viewed the progress of labour as prolonged and they experienced **l**abour pain more negatively compared to women with normal labour. In the group of women with normal labour, women with oxytocin augmentation reported significantly longer lengths (measured in hours) compared to women without oxytocin augmentation. They also perceived their progress of labour as slow although the progress of labour was not diagnosed as prolonged. The pain experience did not differ between the two groups of women with normal labour.

**Table 2:** Self reported length of labour and pain in women following prolonged labour with and without diagnosis and normal labour with and without oxytocin augmentation

	**Prolonged labour**				**Normal labour**		
	**With diagnosis**	P-value*	**Without diagnosis**	P-value**	**Without oxytocin-augmentation**	P-value***	**With oxytocin-augmentation**
	**n = 113**		**n = 67**		**n = 472**		**n = 177**
	**Mean (SD)**		**Mean (SD)**		**Mean (SD)**		**Mean (SD)**
**Length of labour/birth mean hours (SD)**	23.09 (±24.95)	0.00	13.95 (±8.3)	0.000	7.34 (±8.77)	0.015	10.17 (±10.5)
**Experienced labour length (0 = Prolonged- 7 = Rapid)**	20.29 (22.6)	0.00	30.52 (21.0)	0.00	47.07 (21.64)	0.00	38.77 (22.3)
**Pain experience (0 = Very positive- 7 = Very negative)**	4.3 (1.7)	0.91	4.3 (1.4)	0.00	3.6 (1.47)	0.078	3.82 (1.64)

*Prolonged labour with diagnosis versus without diagnosis.

**Prolonged labour with diagnosis or without diagnosis versus Normal labour with and without oxytocin augmentation.

***Normal labour without oxytocin augmentation versus Normal labour with oxytocin augmentation.

Table 3 shows labour outcome in the four groups of women. Prolonged labour with diagnosis was associated with more epidurals, instrumental vaginal births and Caesarean sections. There was no difference in self reported birth complications between women with and without diagnosis of prolonged labour. The numbers of women having a negative birth experience was higher in the group of prolonged labour with diagnosis when compared to women without diagnosis. In the comparison between women following a normal a labour (with and without oxytocin augmentation), significant differences were seen in the use of epidurals and instrumental vaginal births. Women with a normal labour who were treated with oxytocin augmentation reported more often a negative birth experience than did women with a normal labour without oxytocin augmentation. A post hoc analysis, showed that in the group of normal labour 11.7% used an epidural without receiving any augmentation, 16.9% received augmentation without an epidural, and 10% in this group both had an epidural and received augmentation. All analyses were adjusted for parity.

**Table 3:** Labour and birth outcome in women with prolonged labour with and without diagnosis and in women with normal labour with and without oxytocin augmentation

	**Prolonged labour**^**a **^**without diagnosis**	**Prolonged labour**^**a **^**with diagnosis**	**Crude odds ratio**	**Adjusted odds ratio**^**c**^	**Normal labour**^**a **^**without oxytocin augmentation**	**Normal labour**^**a **^**with oxytocin augmentation**	**Crude odds ratio**	**Adjusted odds ratio**^**c**^
	**n = 67**	**n = 113**			**n = 472**	**n = 177**		
	**n (%)**	**n (%)**	**(95% CI)**	**(95% CI)**	**n (%)**	**n (%)**	**(95% CI)**	**(95% CI)**
**Labour augmentation**								
Yes	62 (92.5)	105 (92.9)	0.5 (0.3–3.02)	0.99 (0.31-3.2)	0	177 (100)	Not calculated	
No	5 (7.5)	8 (7.1)	1.0 Ref.^b^	1.0 Ref.	472 (100)	0		
**Epidural**								
Yes	28 (41.8)	88 (77.9)	4.9 (2.5-9.5)***	5.1 (2.6-9.91)***	76 (16.1)	67 *(*37.9)	1.5 (1.3-1.73)***	2.4 (1.6-3.6)***
No	39 (58.2)	25 (22.1)	1.0 Ref.	1.0 Ref.	396 (83.9)	110 (62.1)	1.0 Ref	1.0 Ref
**Mode of delivery**								
Vaginal birth	44 (65.7)	35 (31.0)	1.0 Ref.	1.0 Ref.	444 (94.1)	149 (84.2)	1.0 Ref.	1.0 Ref.
Instrumental vaginal	13 (19.4)	38 (33.6)	3.6 (1.7-7.9)***	3.7 (1.7-8.07)***	14 (3.0)	18 (10.2)	3.83 (1.9-7.9)***	2.81 (1.33-5.94)**
Caesarean section	10 (14.9)	40 (35.4)	5.03 (2.21-11.5)***	5.2 (2.23-11.9)***	14 (3.0)	10 (5.6)	2.3(0.93-4.)	2.0 (0.9-4.73)
**Self reported complications**								
Yes	22 (34.4)	53 (48.2)	1.8 (0.94-3.4)	1.84 (0.97-3.5)	87 (18.9)	40 (23.3)	1.1 (0.95-1.23)	1.1 (0.77-1.66)
No	42 (65.6)	57 (51.8)	1.0 Ref.	1.0 Ref.	374 (73.9)	132 (76.7)	1.0 Ref.	1.0 Ref
**Birth experience**								
Positive	66 (98.5)	91 (80.5)	1.0 Ref.	1.0 Ref.	460 (98.1)	166 (93.8)	1.0 Ref.	1.0 Ref.
Negative	1 (1.5)	22 (19.5)	15.96 (2.1-121.4)***	16.03 (2.11-122.0)***	9 (1.9)	11 (6.2)	3.4 (1.4-8.2)**	2.8 (1.12-7.23)**

^a^Number may not add to 100% due to missing internal values.

^b^Adjusted for number of children.

^c^Reference = Women not exposed to the studied variable.

**p < 0.01., ***p < 0.001.

In Table 4, women’s feelings and experiences during the birth are shown. Women with prolonged labour more often reported that they almost went into a panic during birth, that pain relief saved them, that the difficulties marked them for life, and that it was painful to give birth. In addition, they also strongly agreed with the statement that the worst thing was that they were not able to decide mode of birth and feared that the baby would be damaged during birth. In addition, they agreed more often with the statement that the birth experience made them not want any more children in the future. They less often agreed with the statement that it was exciting to give birth and a prolonged labour was significantly associated with a less positive birth experience. No differences were found in the statements that giving birth was a peak experience and that having children is the meaning of life. When adjusted for parity, the same variables remained statistically significant.

**Table 4:** Women’s feelings and experiences during labour and birth with prolonged labour or normal labour

	**Prolonged labour**^**a, c**^	**Normal labour**^**b, c**^	**Crude odds ratio**	**Adjusted odds ratio**^**d**^
	**n = 180**	**n = 649**		
	**n = (%)**	**n = (%)**	**(95% CI)**	**(95%CI)**
**It was exciting to give birth**				
Strongly agree	139 (77)	563 (89)	1.0 Ref^e^	1.0 Ref
Not strongly agree	40 (23)	69 (11)	0.5 (0.4-0.8)***	0.5 (0.32-0.7)***
**I almost went into panic since I didn’t know what was happening**				
Strongly agree	36 (21)	63 (10)	3.5 (1.9-6.1)***	4.0 (2.13-7.3)***
Not strongly agree	139 (79)	567 (90)	1.0 Ref	1.0 Ref
**Pain relief during birth saved me**				
Strongly agree	99 (57)	239 (39)	2.0 (1.4-2.9***	1.8 (1.2-2.7)**
Not strongly agree	76 (43)	377 (61)	1.0 Ref	Ref
**My difficulties during birth marked me for life**				
Strongly agree	28 (16)	22 (4)	7.0 (2.7-17.7)***	7.2 (2.6-19.6)***
Not strongly agree	144 (84)	581 (96)	1.0 Ref	1.0 Ref
**It was painful to give birth**				
Strongly agree	53 (30)	98 (16)	2.0 (1.2-3.3)**	2.0 (1.12-3.4)**
Not strongly agree	123 (70)	531 (84)	1.0 Ref	1.0 Ref
**It was best to do what the staff told me to**				
Strongly agree	118 (67)	374 (60)	1.2 (0.82-1.7)	1.0 (0.7-1.5)
Not strongly agree	58 (33)	250 (40)	1.0 Ref	1.0 Ref
**The worst was not to be able to decide mode of delivery**				
Strongly agree	25 (16)	29 (5)	3.0 (1.3-6.6)**	3.0 (1.3-7.2)**
Not strongly agree	133 (84)	530 (95)	1.0 Ref	1.0 Ref
**I was frightened that the baby would be damaged during birth**				
Strongly agree	63 (36)	145 (24)	2.0 (1.3-3.2)**	1.8 (1.1-2.8)**
Not strongly agree	110 (64)	471 (76)	1.0 Ref	1.0 Ref
**Giving birth is one of my peak experiences**				
Strongly agree	174 (97)	631 (98)	1.0 (0.6-1.8)	1.1 (0.6-2.0)
Not strongly agree	6 (3)	14 (2)	1.0 Ref	1.0 Ref
**Having children is the meaning of life**				
Strongly agree	147 (84)	575 (90)	0.9 (0.7-1.4)	1.1 (0.8-1.6)
Not strongly agree	28 (16)	62 (10)	1.0 Ref	1.0 Ref
**My birth experience made me decide not to have any more children**				
Strongly agree	19 (11)	23 (4)	3.7 (1.6-8.7)**	4.2 (1.7-10.5)**
Not strongly agree	157 (89)	601 (96)	1.0 Ref	1.0 Ref
**Birth experience**				
Positive	157 (87)	626 (97)	1.0 Ref	1.0 Ref
Negative	23 (13)	20 (3)	4.6 (2.5-8.6)***	3.6 (1.9-7.1)***

^a^Prolonged labour includes women who experienced a prolonged labour with and without diagnosis.

^b^Normal labour includes women who experienced a normal labour with and without oxytocin augmentation.

^c^Number may not add to 100% due to missing internal values.

^d^Adjusted for number of children.

^e^Reference = Women not exposed to the studied variable.

**p < 0.01, ***p < 0.001.

Table 5 presents the results from a logistic regression model of the most important feelings and factors associated with a negative birth experience among women following a prolonged labour (n = 180). The analysis showed that women with a negative birth experience were more likely to be associated with an emergency Caesarean in this group of women with prolonged labour. They agreed with the statements that ‘it was painful in to give birth’ and that ‘my birth experience made me decide not to have any more children’.

**Table 5:** **Most important feelings and factors associated with a negative birth experience among women following a prolonged labour**^**a**^

**Birth experience**		**Positive**	**Negative**	**Adjusted odds ratio**	
		**n = 157 (%)**	**n = 23 (%)**	**OR**	**95% CI**
**Mode of delivery**	**Emergency Caesarean section**	**38 (24)**	**12 (52)**	**9.0**	1.8-45.3**
**Pain experience**	**(0 = Very positive-7 = Very negative)**	**4.13 (1.5)**^**b**^	**5.6 (1.8)**^**b**^	**1.9**	1.2-3.0*
**Women's feelings and experiences**					
**Statement**	**“It was painful to give birth”**				
	**Strongly agree**	**14 (9)**	**12 (52)**	**6.1**	1.2-30.3**
	**“My birth experience made me decide not to have any more children”**				
	**Strongly agree**	**3 (2)**	**8 (35)**	**41.3**	4.9-349.6**
**Adjusted for age, country of birth, marital status, level of education and number of children**					

^a^Prolonged labour includes women who experienced a prolonged labour with and without diagnosis.

^b^Mean (SD).

*p < 0.05, **p < 0.01.

Table 6 presents the results from a logistic regression model of the most important feelings associated with a negative birth experience among women following a normal labour (n = 649). Women with a normal labour who had a negative birth experience agreed significantly less often with the statement that ‘it was exciting to give birth’ and agreed more often with the statement that ‘it was painful in to give birth’ than did women with a positive birth experience.

**Table 6:** **Most important feelings associated with a negative birth experience among women following a normal labour**^**a**^

**Birth experience**		**Positive**	**Negative**	**Adjusted odds ratio**	
		**n = 626 (%)**	**n = 20 (%)**	**OR**	**95% CI**
**Women’s feelings and experience**					
**Statement**	**“It was exiting to give birth”**				
	**Strongly agree**	**566 (93)**	**10 (50)**	**0.13**	0.034-0.5***
	**“It was painful to give birth”**				
	**Strongly agree**	**42 (7)**	**9 (47)**	**7.04**	2.2-22.4***
**Adjusted for age, country of birth, marital status, level of education and number of children**					

^a^Normal labour includes women who experienced a normal labour with and without oxytocin augmentation.

***p < 0.001.

## Discussion

The major findings of this study were that more than every fifth woman was diagnosed with prolonged labour either according to ICD 10 [19] or by a partogram showing a slower progress less than 1 cm per hour. There was also an inappropriate use of oxytocin augmentation among the groups. Approximately every third women in the group of normal labour received oxytocin. For women belonging to the prolonged labour group 7% did not receive oxytocin for labour augmentation.

Women with prolonged labour consisted of more primiparae and had a worse labour outcome and less positive experiences of birth. The prevalence of prolonged labour were among primiparous women 35.6% and among multiparaous women 10.2%, which is fairly similar prevalence that Kjaergaard (2009) reported in her study, where 37% of first-time mothers were diagnosed with prolonged labour [10] and in the study by Selin (2009) the prevalence was 33% in first-time mothers and 7% in women with previous children [9]. In this study, based on data from hospital births, the condition of prolonged labour, although not well diagnosed, is seldom life-threatening. However, it must be noted that worldwide obstructed labour is the cause for maternal mortality in 8% of cases [34].

The result of women’s negative birth experience two months after birth was that they agreed with the statement that their birth experience had made them to decide not to have any more children. Maybe this could reflect a negative attitude towards having more children although two months after birth is a very early timeframe regarding future pregnancies. Still the negative birth experience can make the woman associate birth with fear for future pregnancy and childbirth [35].

In this study more than 20% of women were defined as experiencing prolonged labour based on either the partogram or the ICD10 classification. We also found that 27% of women belonging to the group ‘normal labour’ were exposed to oxytocin augmentation, which should be the treatment for prolonged labour [9,36]. The finding of this high use could be viewed as a misuse of augmentation, a condition previously described by Bernitz et al. (2013), where 42% of women exposed to oxytocin augmentation did not fulfill the criteria for prolonged labour [37]. Generally women used an epidural to a high extent in both the prolonged and normal groups. A post hoc analysis, showed that women used an epidural to a high extent also in the normal groups. These findings suggest that the high use of epidural within the group of normal labour could be a consequence of being exposed to unnecessary interventions and treatments of oxytocin augmentation, to speed up the progress of labour. However the benefit for the woman is being relived from pain, but the use of epidural is also associated with an increased risk for instrumental delivery and emergency cesarean section [38].

The paradox that healthy women received treatment for prolonged labour and women with prolonged labour sometimes (14.5%) were not treated could be viewed as an inappropriate use of oxytocin (affecting 40%) that should be noticed and dealt with regarding identification, classification and treatment of prolonged labour. Misuse of oxytocin in obstetric care is sparsely studied, but it should be noted that Jonsson (2007) in her study found remarkable mistakes according misuse of oxytocin during labour [39]. Most obstetric wards have written guidelines about oxytocin augmentation [40], but fewer guidelines for identifying prolonged labour. The use of partogram or ICD-code is fraught with difficulties in accurate assessment with a true prolonged labour because they are based on the Friedman standards. The consortium of safe labour data [41] are now accepted and has been highlighted by the American college of obstetricians and gynecologist to revise definitions of normal labour progress and to introduce new guidelines for evidence based labour care.

Labour outcome in this study was similar to other studies regarding longer labours [37], more instrumental vaginal births and emergency Caesarean sections [10,21,42]. When dealing with issues related to obstetric care, e.g. prolonged labour, which is a common obstetric diagnosis, women’s feelings and experiences must be taken into account. As it was shown that these more ‘soft variables’ had a strong impact on women’s experiences of the length labour and emergency caeserean as well as their view towards future reproduction two months after birth. Women with prolonged labour were, in addition, more likely to have a negative birth experience. It is known that a negative birth experience is associated with a delay in subsequent births [35], fear of birth [43], a preference for Caesarean section in future births [44] and maternal ill-health [45].

The only background variable that differed between women diagnosed with prolonged labour or not was parity, with more primiparas subjected to prolonged labour, a finding similar to other studies [9,46]. Other well-known characteristics of women with prolonged labour, such as high body mass index [44] or high maternal age [47], were not confirmed in the present study.

This study is compromised by its observational design, the regional context and the exclusion of non-Swedish speaking women, which makes it difficult to generalize the findings to all women. Another weakness is that some of the significant factors associated with a negative birth experience among women with a prolonged labour gave an odds ratio with wide confidence intervals. The wide values of the confidence intervals are a cause for concern and should be interpreted with caution. Strength of the study is the fairly large sample and the ability to combine results from birth records and questionnaires, which makes it possible to present obstetric outcome together with women’s experiences.

Using the social security numbers given to all Swedish citizens at birth affords the opportunity to obtain obstetric data from medical records. The identification and classification of prolonged labour was based on birth records and checked manually.

Another strength is that the sample characteristics did not deviate from the pregnant population in the region.

## Conclusion

Prolonged labour is a complicated condition negatively affecting obstetric outcome and women’s experiences. There is a need for consensus in classification and treatment of prolonged labour. Careful management of interventions is crucial in order to keep normal births normal and avoid mistreatment. Increased clinical skill and a good documentation of the progress of labour in birth records, are of great importance to identify and classify prolonged labour, in order to improve care for all women and their experiences of birthing processes regardless of whether they experience prolonged labour or not.

## Competing interests

The authors declare that they have no competing interests.

## Authors’ contributions

AN, IH designed the study and undertook data collection. AN participated in the study design, performed data edits and statistical analyses, wrote the draft, and reviewed and finalized the manuscript. IH participated in the study design, performed data edits, statistical analyses and edited and reviewed the final manuscript. Both authors read and approved the final manuscript.

## Pre-publication history

The pre-publication history for this paper can be accessed here:

http://www.biomedcentral.com/1471-2393/14/233/prepub
